# Complex Mechanisms Underlying the Coexistence of Diabetic Complications, Chronic Kidney Disease, and Hemorrhagic Stroke: A Case Report

**DOI:** 10.7759/cureus.95844

**Published:** 2025-10-31

**Authors:** Sandra Czyz, Wiktoria Marszal, Oliwia Sysło, Nina Saracen

**Affiliations:** 1 Medical Education, Academy of Silesia, Katowice, POL; 2 Faculty of Medicine, Academy of Silesia, Katowice, POL

**Keywords:** brain ct scan, chronic kidney disease (ckd), diabetes foot ulcer, diabetes type 2, foot phlegmon, neuropsychiatric manifestations, stroke

## Abstract

Diabetes mellitus type 2 is a chronic metabolic disease that presents a growing global health challenge. Its long-term complications, including nephropathy, neuropathy, and retinopathy, significantly contribute to patient morbidity and the healthcare burden. Diabetic kidney disease, one of the significant complications, often progresses to end-stage renal failure, necessitating renal replacement therapy. This case report describes a 67-year-old man with longstanding type 2 diabetes mellitus and hypertension complicated by progressive chronic kidney disease, diabetic foot syndrome, and subsequent neurological and psychiatric manifestations.

The patient was initially admitted to the nephrology department for predialysis assessment and optimization of anemia management. Over the following months, he experienced recurrent hospitalizations due to diabetic foot ulceration, phlegmon requiring surgical intervention, and episodes of psychotic syndrome related to metabolic and vascular factors.

The final admission to the stroke unit revealed a left thalamic intracerebral hemorrhage associated with severe arterial hypertension and worsening disturbances of consciousness. A multidisciplinary approach was employed, including nephrological, surgical, psychiatric, and neurological care, along with complex polypharmacotherapy and insulin therapy.

This case illustrates the intricate relationship between diabetes, chronic kidney disease, and cerebrovascular complications. It emphasizes the importance of early nephrological and metabolic monitoring, personalized treatment strategies, and ongoing interdisciplinary collaboration. Early recognition and management of diabetic complications can significantly improve prognosis and prevent life-threatening outcomes in patients with advanced type 2 diabetes mellitus.

## Introduction

Diabetes is a persistent, progressive cardiometabolic disorder that is a leading cause of illness, disability, and death worldwide. Managing it effectively requires a comprehensive, person-centered approach, including blood sugar regulation, control of cardiovascular risk factors such as hyperlipidaemia, hypertension, and tobacco use, maintaining a healthy weight, early detection and treatment of microvascular, macrovascular, and metabolic complications, addressing mental health, reducing treatment burden, tackling social determinants of health, and enhancing overall quality of life [1].

Over the past decade, significant improvements have been made across all areas of diabetes care. The disease impacts daily functioning and quality of life, causing considerable morbidity and early death. Recently, concerns have been raised that over a third of diabetes-related deaths occur in people under 60. This trend is mainly due to increased intake of unhealthy foods and sedentary lifestyles, which lead to higher BMI and fasting plasma glucose levels. Notably, higher BMI is strongly associated with type 2 diabetes. The aging population also influences this pattern, as diabetes is more common among older adults [2].

Inadequate diabetes management can lead to diabetic foot ulcers (DFUs), which typically appear as ulcers on the sole. These are full-thickness wounds in the dermis, usually in weight-bearing or exposed areas of the lower leg, particularly below the ankle. DFUs often result in more extended hospital stays and higher healthcare expenses. Hence, early detection and targeted treatment are essential for this common complication of diabetes. Combining standard local care with innovative methods like stem cell therapy can decrease morbidity, reduce amputation risk, and prevent death from DFU. Research is ongoing into new treatment options to further alleviate the healthcare burden associated with DFU [3].

A stroke is a severe nervous system disorder, the essence of which is a disturbance in the blood flow within the brain. This can lead to the formation of clots that block arteries or damage blood vessels, resulting in intracranial bleeding. When the blood and oxygen supply to the nerve cells is interrupted, they begin to die. A consequence of a stroke may also be cognitive dysfunction, the development of dementia, and the onset of depression [4].

Stroke is a condition that most often occurs in older people, but the risk of its occurrence also applies to younger people. In 2009, as many as 34% of patients hospitalized due to stroke were under 65 years old. The frequency of this condition also varies depending on race and ethnicity, which affects the observed epidemiological trends [5].

High blood pressure (BP) remains the top global risk factor for cardiovascular disease (CVD) morbidity and mortality. While lowering BP can reduce the risk of CVD events and deaths, the most effective approach focuses on preventing high BP initially, along with detecting, treating, and managing hypertension. The first step in managing hypertension is obtaining an accurate diagnosis [6].

## Case presentation

Nephrology admission: December 13-15, 2023

The patient was admitted to the nephrology department on December 13, 2023, due to worsening chronic type 2 diabetes. Diabetes is a long-term and progressive cardiometabolic disorder, and it is a significant cause of morbidity, disability, and death worldwide.

The goals were to begin predialysis, evaluate possible adjustments to anemia management, review the virological profile, and determine the safest approach for potential renal replacement therapy. The patient's medical history includes type 2 diabetes and hypertension, both lasting over 20 years. This patient has experienced various diabetes related complications, including retinopathy, polyneuropathy, and worsening nephropathy with significant proteinuria. They also have a history of diabetic foot syndrome, a severe complication resulting from poorly controlled long-term diabetes, often manifesting as foot ulcers. Recently, the patient developed a wound on the right foot during treatment, the day before admission, and is currently on antibiotics for a diabetic foot infection.

Additionally, they have prostatic hyperplasia but no history of heart disease or chest pain.

In 2022, he was diagnosed twice in the Emergency Department with suspected ischemia of the central nervous system (CNS). During his stay at the Emergency Department, the above suspicions were ruled out. In the study results, there was a decrease in glomerular filtration rate (GFR) of 1.5 g/dL over the biennium, resulting in a reduction from 36 to 16 ml/min. It is accompanied by the above-mentioned proteinuria with an albumin-to-creatinine ratio (ACR) of 1600, acid-base imbalance, increased parathyroid hormone (PTH) levels, and anemia. Based on the overall clinical picture, it was decided to initiate preparations for renal replacement treatment using the selected hemodialysis method.

A referral was issued to the Surgical Department to produce an arteriovenous fistula for hemodialysis. The patient remains under the care of ophthalmologists, urologists, and endocrinologists, also known as diabetologists. In the treatment of two-type diabetes, the patient uses insulin therapy, which recommends Gensulin N and Gensulin R. The patient's injections are administered several times a day, except for a long-acting preparation. A diabetes consultation is advisable.

In-Hospital Management

The ward received treatment in the form of: insulin therapy, antibiotic 625 mg, Controloc 20 mg, Betaserc forte, Vicebrol, Dipperamm, Finaster 5 mg, Omnic 0.4, Trifas 10 mg, Nemovit, Polocard 75 mg, Tulipi 20 mg, Nebilet 5 mg, Silamil 10 mg, amlodipine 5 mg, valsartan 160 mg, and hydrochlorothiazide 12.5 mg.

Post-discharge Pharmacotherapy

The patient was discharged on December 15, 2023, in good general condition with the following recommendations: dietary recommendations were issued in the form of an antidiabetic diet, further treatment in the form of insulin therapy (insulin Gensulin R and N) in accordance with diabetes recommendations. Table 1 presents the medications that were prescribed to the patient upon discharge from the ward.

**Table 1:** Recommended medications after discharge from the nephrology department

Drug	Dosage	Time of administration
Controloc 20 mg	Once daily	Morning
Finaster 5 mg	Once daily	Morning
Omnic 0.4	Once daily	Morning
Trifas 10 mg	Once daily	Morning
Polocard 75 mg	Once daily	Morning
Tulip 20 mg	Once daily	Morning
Nebilet 5 mg	Once daily	Morning
Silamil 10 mg	½ tablet once daily	Overnight
Amlodipine 5 mg	Once daily	Morning
Valsartan 160 mg	Once daily	Morning
Diuver 10 mg	Once daily	Morning

Periodic inspection at the Nephrology Clinic was also recommended, as well as further outpatient treatment and antibiotic therapy, as recommended by the surgeon. A referral was issued to produce a thetan fistula for hemodialysis and a referral to the Urology Clinic.

Surgical admission: July 23-28, 2025

In July, the patient was admitted to the Department of Surgery for excision of a right foot ulcer. Both surgical and pharmacological treatments were administered.

Surgical admission: August 22-September 5, 2025

In August, the patient was hospitalized in the Department of General Surgery due to a phlegmon of the right foot. A combination of surgical and pharmacological management was implemented, yielding a favorable outcome. During hospitalization, the patient exhibited symptoms consistent with a psychotic syndrome, particularly at night, presenting with disorientation and episodes of aggression.

Psychiatric consultation: August 22, 2025

On August 22, 2025, the patient attended a psychiatric consultation. He was transported by medical services and left in the psychiatric department's examination room. The patient was in critical condition, collapsing onto a couch supported by his wife. He had multiple health issues, including generalized atherosclerosis, previous CNS ischemic events, and overall weakness. He was exhausted, highly drowsy, exhibited incoherent behavior, and occasionally vomited. His condition prevented psychiatric hospitalization. During a nighttime assessment, he experienced insomnia, agitation, and aggression, characteristic of "sunset syndrome," which involves the emergence or worsening of neuropsychiatric symptoms (NPS) during late afternoon or early evening. This syndrome is well known among healthcare providers caring for people with dementia [7]. The diagnosis was symptomatic disturbances of consciousness caused by somatic conditions. The patient was advised to visit the Emergency Department because of his severe overall state, to take haloperidol 5 mg at bedtime, and if there was no improvement, to add Tiaprid PCMS 100 mg, one tablet. Outpatient treatment will continue with the Environmental Treatment Team.

Stroke unit admission: September 10-23, 2025

In September 2025, a 67-year-old male patient with a history of chronic kidney disease of diabetic etiology, under regular nephrological supervision, was admitted to the stroke unit due to progressive disturbances of consciousness persisting for several hours, accompanied by episodes of aggressive behavior. His medical history included type 2 diabetes mellitus requiring insulin therapy, arterial hypertension, and benign prostatic hyperplasia. The patient had an arteriovenous fistula located on the left forearm; however, at the time of admission, he was not undergoing dialysis.

**Figure 1:**
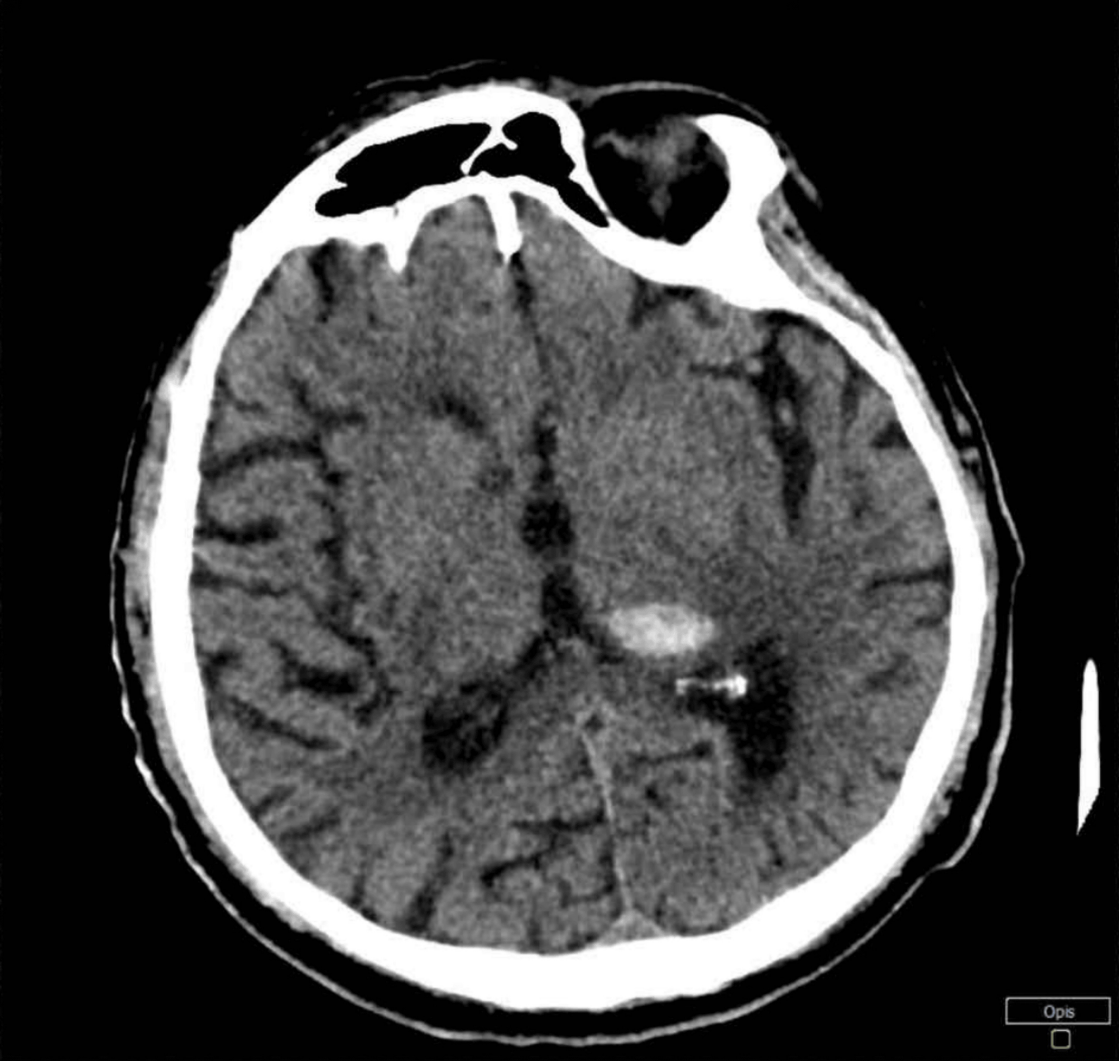
CT scan of the brain

Cranial computed tomography (CT) performed at admission revealed an acute intracerebral hematoma localized in the left thalamus, measuring 20 × 11 mm. The lesion caused a mild mass effect, manifesting as a rightward displacement of brain structures by approximately 5 mm (Figure 1).

Following neurological consultation, neurosurgical intervention was deemed unnecessary. Neurological examination revealed features of psychosis with disturbances of allo- and autopsychic contact, as well as a trace right-sided hemiparesis. Further diagnostics included ultrasonography of the common and internal carotid arteries and vertebral arteries, which demonstrated normal vessel diameter, preserved patency, and no hemodynamically significant stenoses. Ambulatory blood pressure monitoring by Holter monitor revealed markedly elevated blood pressure, which was effectively reduced during hospitalization.

A follow-up cranial CT scan demonstrated the persistence of the left thalamic hematoma with unchanged dimensions, no mass effect, and signs of partial hemolysis. Additionally, a 9 mm hypodense lesion was observed in the right frontal lobe at the cortico-subcortical junction, interpreted as a vascular-related focus. No other focal intracerebral pathologies were identified. During hospitalization, the patient also developed symptoms of a urinary tract infection.

Neurological examination at discharge revealed persistent disturbances of consciousness and psychotic features, without cranial nerve deficits or limb paresis. The patient was diagnosed with a hemorrhagic stroke, presenting with auto- and allopsychic disorientation and episodes of aggression. Partial regression of neurological and psychotic symptoms was noted during hospitalization.

In-Hospital Management

Management in the ward included polypharmacotherapy, comprising antibiotic therapy (amoxicillin-clavulanate, ciprofloxacin), antihypertensive therapy (nebivolol, nitrendipine, doxazosin, moxonidine), diuretics (furosemide), a statin (tulip), antipsychotic medications (haloperidol, tiapride, quetiapine), insulin therapy, supportive care with intravenous fluids and mineral-electrolyte solutions, proton pump inhibitors (pantoprazole), analgesics and antipyretics (paracetamol), anticoagulants (enoxaparin), urological medications (finasteride, tamsulosin), and a probiotic (trilac). Additionally, clonazepam was used for anticonvulsant and sedative purposes.

After hospitalization, the patient was transferred to a long-term care facility. A diabetic diet with a restricted fat content, regular monitoring of blood glucose and blood pressure twice daily, and prompt consultation with a primary care physician in the event of abnormal readings, was recommended.

Post-discharge Pharmacotherapy

After discharge, the patient continued multidrug therapy, including gastroprotective, antihypertensive, antidiabetic, psychiatric, and anti-infective treatment. Insulin therapy and iron supplementation were maintained, along with recommendations for adequate hydration (approximately 2,000 mL of oral fluids daily). Table 2 presents the medications that were prescribed to the patient upon discharge from the ward.

**Table 2:** Recommended medications after discharge from the stroke unit SR: sustained release; s.c.: subcutaneous

Drug	Dosage	Time of administration
Pantoprazole 20 mg	Once daily	Morning
Zahron 20 mg	Once daily	Evening
Nebivolol 5 mg	Once daily	Morning
Moxonidine 20 mg	Twice daily	Morning and evening
Nitrendipine 10 mg	Twice daily	Morning and evening
Joxar 1 mg	Once daily	Morning
Forxiga 10 mg	Once daily	Morning
Finasteride 5 mg	Once daily	Morning
Tiapride 100 mg	½ tablet twice daily	Morning and evening
Iron SR	Twice daily	Morning and evening
Ciprofloxacin 500 mg	Twice daily	Morning and evening
Insulin aspart with protamine (NovoMix 50) 12 units s.c.	Once daily	Morning

**Figure 2:**
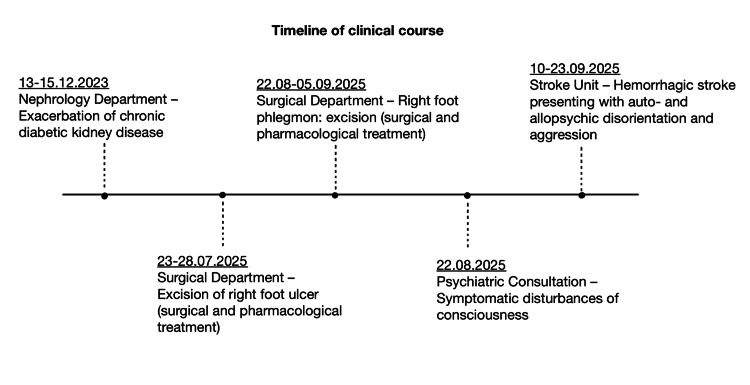
Timeline of clinical course

The timeline of the patient’s clinical course is presented in Figure 2. The patient was admitted to the ward four times between December 13, 2023, and September 23, 2025.

## Discussion

This case illustrates the complexity of managing a patient with multimorbidity, where long-standing type 2 diabetes mellitus, complicated by nephropathy, retinopathy, polyneuropathy, and diabetic foot syndrome, has significantly impaired quality of life and led to the progression of advanced chronic kidney disease (CKD) [1,2]. Despite insulin therapy and supportive nephrological management, renal function declined with a marked reduction in GFR, necessitating preparation for renal replacement therapy. The creation of an arteriovenous fistula and nephrology consultations highlight the importance of early planning for dialysis in accordance with current nephrology guidelines [8].

The development of an intracerebral hemorrhage in the left thalamus was most likely multifactorial, with arterial hypertension, chronic kidney disease, and diabetic vasculopathy serving as major risk factors. Hypertension remains the leading cause of intracerebral hemorrhage, and in patients with diabetes and CKD, blood pressure control is often challenging due to fluid imbalance, vascular stiffness, and long-standing metabolic disturbances. In this case, ambulatory blood pressure monitoring confirmed poorly controlled hypertension, which likely contributed to the hemorrhagic event [4,9].

In addition, the occurrence of “sundowning syndrome” emphasized the need for close collaboration between neurology, psychiatry, and the caregiving team to stabilize the patient’s mental status [6].

Another major therapeutic challenge was the presence of recurrent infections, including urinary tract infections and chronic infection associated with diabetic foot syndrome. The coexistence of CKD, diabetes-related immune dysfunction, and polypharmacy significantly increased susceptibility to infections and sepsis, necessitating repeated antibiotic therapies [3]. This underscores the importance of infection surveillance and tailored antimicrobial management in such high-risk patients.

Overall, this case highlights the interplay of metabolic, vascular, infectious, and psychiatric complications in advanced diabetes. It demonstrates the need for an individualized, multidisciplinary approach involving nephrology, neurology, psychiatry, diabetology, and surgery. Comprehensive and coordinated care is essential not only for stabilizing acute conditions, such as stroke or infection, but also for long-term management of CKD and neuropsychiatric sequelae. Early dialysis planning, strict infection control, and continuous psychiatric support remain crucial for optimizing prognosis and maintaining quality of life in patients with advanced diabetes and systemic complications.

## Conclusions

This case demonstrates the complex, multifactorial nature of complications in patients with long-term type 2 diabetes mellitus, involving interactions between metabolic, vascular, infectious, and psychiatric disorders. The coexistence of diabetic nephropathy, chronic kidney disease, and cerebrovascular events reflects the progressive, systemic effects of poorly controlled metabolic disease, which in this case resulted in a hemorrhagic stroke. Effective management of such a complex condition requires a multidisciplinary approach involving nephrology, endocrinology (diabetology), neurology, psychiatry, and surgery to address both acute issues and long-term health concerns simultaneously. Early referral to a nephrologist, strict control of blood sugar and blood pressure, prompt treatment of infections, and timely preparation for renal replacement therapy-including the creation of vascular access-are critically important. This case emphasizes that personalized care, close medical monitoring, and interdisciplinary collaboration can significantly reduce complications, enhance quality of life, and extend survival in patients with advanced type 2 diabetes mellitus.
